# Crystal structure of 4-benzamido-2-hy­droxy­benzoic acid

**DOI:** 10.1107/S2056989015009032

**Published:** 2015-05-20

**Authors:** Muhammad Shahid, Muhammad Aziz Choudhary, Arshad Farooq Butt, Muhammad Nawaz Tahir, Muhammad Salim

**Affiliations:** a Department of Chemistry, University of the Punjab, Lahore, Punjab, Pakistan; b Department of Chemistry, Mirpur University of Science and Technology (MUST), Mirpur, Azad Jammu and Kashmir, Pakistan; c Department of Physics, University of Sargodha, Sargodha, Punjab, Pakistan

**Keywords:** crystal structure, hydrogen bonding, π–π inter­actions

## Abstract

In the title compound, C_14_H_11_NO_4_, the dihedral angle between the mean planes of the aromatic rings is 3.96 (12)° and an intra­molecular O—H⋯O hydrogen bond closes an *S*(6) ring. A short intra­molecular C—H⋯O contact is also seen. In the crystal, carb­oxy­lic acid inversion dimers linked by pairs of O—H⋯O hydrogen bonds generate *R*_2_^2^(8) loops. Conversely, the N—H group does not form a hydrogen bond. Aromatic π–π inter­actions exist at a centroid–centroid distance of 3.8423 (15) Å between the benzene rings. An extremely weak C—H⋯π inter­action also is present.

## Related literature   

For related structures, see: Gibson *et al.* (2010 ▸); Júnior *et al.* (2013 ▸); Montis & Hursthouse (2012 ▸).
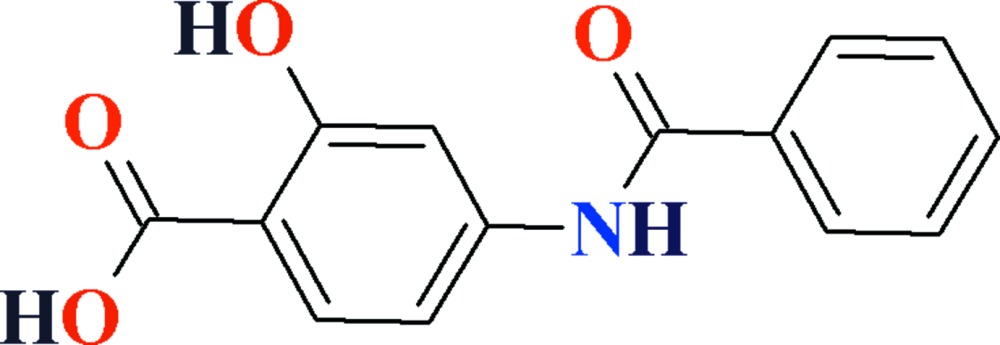


## Experimental   

### Crystal data  

C_14_H_11_NO_4_
*M* *_r_* = 257.24
Monoclinic, 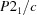

*a* = 5.6689 (5) Å
*b* = 32.039 (3) Å
*c* = 6.6413 (5) Å
β = 103.530 (5)°
*V* = 1172.74 (18) Å^3^
*Z* = 4
Mo *K*α radiation
μ = 0.11 mm^−1^
*T* = 296 K
0.38 × 0.30 × 0.16 mm

### Data collection  

Bruker Kappa APEXII CCD diffractometer
Absorption correction: multi-scan (*SADABS*; Bruker, 2005 ▸) *T*_min_ = 0.960, *T*_max_ = 0.984
9560 measured reflections
2582 independent reflections
1602 reflections with *I* > 2σ(*I*)
*R*_int_ = 0.044

### Refinement  

*R*[*F* ^2^ > 2σ(*F* ^2^)] = 0.057
*wR*(*F* ^2^) = 0.147
*S* = 1.04
2582 reflections
174 parameters
H-atom parameters constrained
Δρ_max_ = 0.20 e Å^−3^
Δρ_min_ = −0.22 e Å^−3^

### 

Data collection: *APEX2* (Bruker, 2007 ▸); cell refinement: *SAINT* (Bruker, 2007 ▸); data reduction: *SAINT*; program(s) used to solve structure: *SHELXS97* (Sheldrick, 2008 ▸); program(s) used to refine structure: *SHELXL2014* (Sheldrick, 2015 ▸); molecular graphics: *ORTEP-3 for Windows* (Farrugia, 2012 ▸) and *PLATON* (Spek, 2009 ▸); software used to prepare material for publication: *WinGX* (Farrugia, 2012 ▸) and *PLATON*.

## Supplementary Material

Crystal structure: contains datablock(s) global, I. DOI: 10.1107/S2056989015009032/hb7423sup1.cif

Structure factors: contains datablock(s) I. DOI: 10.1107/S2056989015009032/hb7423Isup2.hkl

Supporting information file. DOI: 10.1107/S2056989015009032/hb7423Isup3.cml

**Fig. 1.**
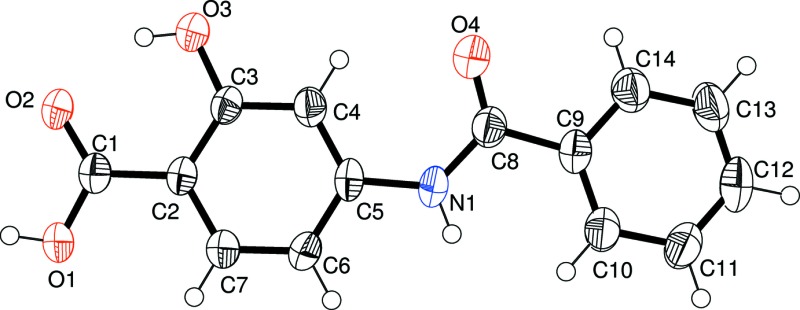
View of the title compound with displacement ellipsoids drawn at the 50% probability level. The dotted line show intramolecular H-bonding.

. DOI: 10.1107/S2056989015009032/hb7423fig1.tif
View of the title compound with displacement ellipsoids drawn at the 50% probability level. The dotted line show intra­molecular H-bonding.

**Fig. 2.**
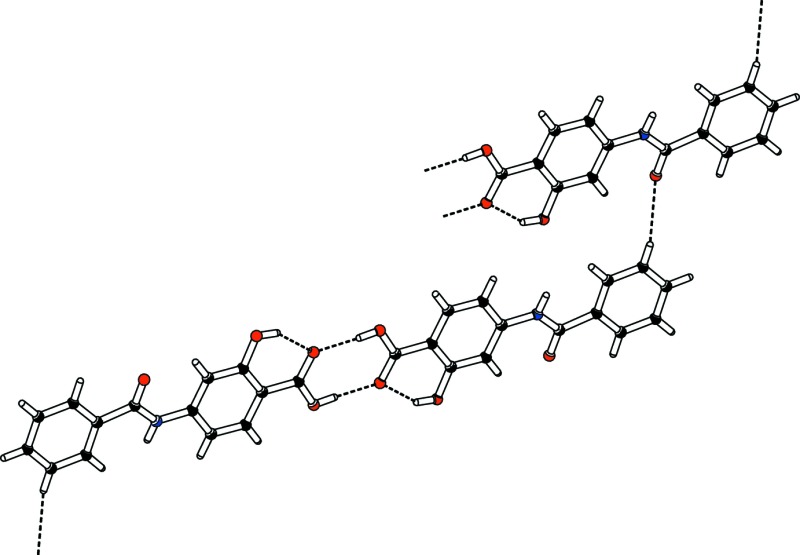
The partial packing, which shows that molecules form dimers and which are interlinked with each othere.

. DOI: 10.1107/S2056989015009032/hb7423fig2.tif
The partial packing, which shows that mol­ecules form dimers and which are inter­linked with each othere.

CCDC reference: 1400009

Additional supporting information:  crystallographic information; 3D view; checkCIF report

## Figures and Tables

**Table 1:** Hydrogen-bond geometry (, ) *Cg*2 is the centroid of the C9C14 ring.

*D*H*A*	*D*H	H*A*	*D* *A*	*D*H*A*
O1H1O2^i^	0.82	1.83	2.6470(19)	176
O3H3O2	0.82	1.88	2.601(2)	146
C4H4O4	0.93	2.23	2.828(3)	122
C12H12*Cg*2^ii^	0.93	2.95	3.773(3)	142

Symmetry codes: (i) 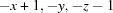; (ii) 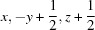.

## supplementary crystallographic information

### S1. Comment

The crystal structures of methyl 4-(isonicotinoylamino)-2-methoxybenzoate (Gibson, 2010), 4-acetamido-2-hydroxybenzoic acid (Montis & Hursthouse, 2012) and 2-([(4-carboxy-3-hydroxyphenyl)iminiumyl]methyl)phenolate (Junior *et al.*, 2013) have been published which are related to the title compound (I, Fig. 1). (I) is synthesized for the biological studies and for the complexation with different metals.

In (I), the parts of 4-aminosalicylic acid *A* (C1–C7/O1/O2/O3) and benzaldehyde *B* (C8—C14/O4) are planar with r. m. s. deviation of 0.0189 Å and 0.0524 Å, respectively. The dihedral angle between A/B is 5.86 (10)°. All heavy atoms of the compound form roughly a plane with r. m. s. devation of 0.0997 Å. In this plane the maximumu deviation is for O4-atom which is 0.321 (2) Å from the mean square plane. There exist intermolecular H-bonding of O—H···O type (Table 1, Fig. 2) forming *S* (6) loop. The molecules are dimerized due to inversion and O—H···O type of H-bonding (Table 1, Fig. 2) completing *R*_2_^2^(8) rings motifs (Table 1, Fig. 2). The dimers are interlinked due to C—H···O interactions (Table 1, Fig. 2). There exist strong π–π interactions at a distance of 3.8423 (15) Å between the centeroids of *Cg*1—*Cg*2^i^ and *Cg*2—*Cg*1^ii^ [i = *x*, *y*, -1 + *z*: ii = *x*, *y*, 1 + *z*], where *Cg*1 and *Cg*2 are the centroids of benzene rings *C* (C2—C7) and *D* (C9—C14), respectively. There also exist a C—H···π interaction (Table 1) and may have important role in stabilizing the molecules.

### S2. Experimental

4-Aminosalicylic acid was dissolved in ethylacetate and equimolar benzoyl chloride was added to the solution under stirring. The mixture was stired for 5 h. Light orange plates were obtained after 48 h.

### S3. Refinement

The H atoms were positioned geometrically (C–H = 0.93 Å, N—H= 0.86 Å, O—H= 0.82 Å) and refined as riding with *U*_iso_(H) = *xU*_eq_ (C, N, O), where *x* = 1.5 for hydroxy and *x* = 1.2 for other H-atoms.

### Crystal data

**Table:**

C_14_H_11_NO_4_	*F*(000) = 536
*M**_r_* = 257.24	*D*_x_ = 1.457 Mg m^−^^3^
Monoclinic, *P*2_1_/*c*	Mo *K*α radiation, λ = 0.71073 Å
*a* = 5.6689 (5) Å	Cell parameters from 1602 reflections
*b* = 32.039 (3) Å	θ = 3.2–27.1°
*c* = 6.6413 (5) Å	µ = 0.11 mm^−^^1^
β = 103.530 (5)°	*T* = 296 K
*V* = 1172.74 (18) Å^3^	Plate, light orange
*Z* = 4	0.38 × 0.30 × 0.16 mm

### Data collection

**Table:**

Bruker Kappa APEXII CCD diffractometer	2582 independent reflections
Radiation source: fine-focus sealed tube	1602 reflections with *I* > 2σ(*I*)
Graphite monochromator	*R*_int_ = 0.044
Detector resolution: 7.70 pixels mm^-1^	θ_max_ = 27.1°, θ_min_ = 3.2°
ω scans	*h* = −7→7
Absorption correction: multi-scan (*SADABS*; Bruker, 2005)	*k* = −41→24
*T*_min_ = 0.960, *T*_max_ = 0.984	*l* = −8→8
9560 measured reflections	

### Refinement

**Table:**

Refinement on *F*^2^	Primary atom site location: structure-invariant direct methods
Least-squares matrix: full	Secondary atom site location: difference Fourier map
*R*[*F*^2^ > 2σ(*F*^2^)] = 0.057	Hydrogen site location: inferred from neighbouring sites
*wR*(*F*^2^) = 0.147	H-atom parameters constrained
*S* = 1.04	*w* = 1/[σ^2^(*F*_o_^2^) + (0.062*P*)^2^ + 0.2313*P*] where *P* = (*F*_o_^2^ + 2*F*_c_^2^)/3
2582 reflections	(Δ/σ)_max_ < 0.001
174 parameters	Δρ_max_ = 0.20 e Å^−^^3^
0 restraints	Δρ_min_ = −0.22 e Å^−^^3^

### Special details

**Table:**

Geometry. All e.s.d.'s (except the e.s.d. in the dihedral angle between two l.s. planes) are estimated using the full covariance matrix. The cell e.s.d.'s are taken into account individually in the estimation of e.s.d.'s in distances, angles and torsion angles; correlations between e.s.d.'s in cell parameters are only used when they are defined by crystal symmetry. An approximate (isotropic) treatment of cell e.s.d.'s is used for estimating e.s.d.'s involving l.s. planes.
Refinement. Refinement of *F*^2^ against ALL reflections. The weighted *R*-factor *wR* and goodness of fit *S* are based on *F*^2^, conventional *R*-factors *R* are based on *F*, with *F* set to zero for negative *F*^2^. The threshold expression of *F*^2^ > σ(*F*^2^) is used only for calculating *R*-factors(gt) *etc*. and is not relevant to the choice of reflections for refinement. *R*-factors based on *F*^2^ are statistically about twice as large as those based on *F*, and *R*-factors based on ALL data will be even larger.

### Fractional atomic coordinates and isotropic or equivalent isotropic displacement parameters (Å^2^)

**Table:**

	*x*	*y*	*z*	*U*_iso_*/*U*_eq_	
O1	0.7252 (3)	0.01068 (6)	−0.2615 (2)	0.0515 (5)	
H1	0.6964	−0.0023	−0.3708	0.077*	
O2	0.3434 (3)	0.03147 (5)	−0.3868 (2)	0.0478 (4)	
O3	0.1427 (3)	0.08122 (6)	−0.1660 (2)	0.0554 (5)	
H3	0.1498	0.0670	−0.2670	0.083*	
O4	0.2383 (3)	0.14573 (7)	0.4823 (3)	0.0723 (6)	
N1	0.6221 (3)	0.12432 (6)	0.4934 (2)	0.0457 (5)	
H1A	0.7682	0.1253	0.5677	0.055*	
C1	0.5318 (4)	0.03152 (7)	−0.2456 (3)	0.0379 (5)	
C2	0.5512 (4)	0.05479 (7)	−0.0538 (3)	0.0339 (5)	
C3	0.3563 (4)	0.07877 (7)	−0.0233 (3)	0.0367 (5)	
C4	0.3735 (4)	0.10154 (7)	0.1581 (3)	0.0415 (6)	
H4	0.2419	0.1170	0.1775	0.050*	
C5	0.5876 (4)	0.10094 (7)	0.3091 (3)	0.0378 (5)	
C6	0.7835 (4)	0.07703 (8)	0.2805 (3)	0.0439 (6)	
H6	0.9273	0.0766	0.3826	0.053*	
C7	0.7651 (4)	0.05425 (7)	0.1036 (3)	0.0416 (6)	
H7	0.8959	0.0382	0.0873	0.050*	
C8	0.4525 (4)	0.14561 (8)	0.5685 (3)	0.0449 (6)	
C9	0.5423 (4)	0.16900 (7)	0.7674 (3)	0.0417 (6)	
C10	0.7710 (5)	0.16503 (9)	0.8943 (3)	0.0550 (7)	
H10	0.8831	0.1472	0.8569	0.066*	
C11	0.8349 (5)	0.18742 (9)	1.0775 (4)	0.0620 (8)	
H11	0.9896	0.1846	1.1626	0.074*	
C12	0.6713 (6)	0.21360 (9)	1.1334 (4)	0.0639 (8)	
H12	0.7149	0.2287	1.2561	0.077*	
C13	0.4429 (5)	0.21765 (9)	1.0090 (4)	0.0648 (8)	
H13	0.3319	0.2356	1.0473	0.078*	
C14	0.3771 (5)	0.19531 (8)	0.8274 (4)	0.0533 (7)	
H14	0.2210	0.1979	0.7446	0.064*	

### Atomic displacement parameters (Å^2^)

**Table:**

	*U*^11^	*U*^22^	*U*^33^	*U*^12^	*U*^13^	*U*^23^
O1	0.0514 (10)	0.0617 (12)	0.0387 (9)	0.0102 (9)	0.0050 (7)	−0.0200 (8)
O2	0.0507 (9)	0.0585 (11)	0.0312 (8)	0.0042 (8)	0.0034 (7)	−0.0140 (7)
O3	0.0487 (10)	0.0754 (14)	0.0379 (9)	0.0111 (9)	0.0016 (7)	−0.0185 (8)
O4	0.0574 (12)	0.1049 (18)	0.0502 (10)	0.0122 (11)	0.0037 (8)	−0.0290 (10)
N1	0.0492 (11)	0.0537 (13)	0.0324 (9)	−0.0019 (10)	0.0059 (8)	−0.0152 (9)
C1	0.0473 (13)	0.0353 (13)	0.0319 (10)	−0.0030 (11)	0.0111 (9)	−0.0027 (9)
C2	0.0411 (12)	0.0335 (13)	0.0275 (10)	−0.0052 (10)	0.0090 (8)	−0.0033 (8)
C3	0.0414 (12)	0.0412 (14)	0.0271 (10)	−0.0043 (10)	0.0072 (8)	−0.0029 (9)
C4	0.0461 (13)	0.0452 (15)	0.0346 (11)	−0.0001 (11)	0.0120 (9)	−0.0068 (10)
C5	0.0511 (13)	0.0366 (14)	0.0276 (10)	−0.0068 (11)	0.0133 (9)	−0.0064 (9)
C6	0.0442 (13)	0.0548 (16)	0.0298 (11)	0.0008 (12)	0.0031 (9)	−0.0073 (10)
C7	0.0440 (13)	0.0462 (15)	0.0345 (11)	0.0025 (11)	0.0090 (9)	−0.0057 (10)
C8	0.0515 (15)	0.0474 (16)	0.0347 (11)	0.0022 (12)	0.0079 (10)	−0.0041 (10)
C9	0.0570 (14)	0.0368 (14)	0.0323 (11)	−0.0025 (11)	0.0124 (10)	−0.0026 (9)
C10	0.0643 (16)	0.0577 (17)	0.0404 (13)	0.0090 (13)	0.0070 (11)	−0.0099 (11)
C11	0.0696 (18)	0.066 (2)	0.0430 (14)	0.0040 (15)	−0.0008 (12)	−0.0123 (13)
C12	0.089 (2)	0.0590 (19)	0.0442 (14)	−0.0075 (16)	0.0173 (14)	−0.0202 (12)
C13	0.0700 (18)	0.064 (2)	0.0639 (17)	0.0005 (15)	0.0223 (14)	−0.0274 (14)
C14	0.0570 (15)	0.0515 (17)	0.0525 (14)	−0.0018 (13)	0.0152 (11)	−0.0103 (12)

### Geometric parameters (Å, º)

**Table:**

O1—C1	1.309 (3)	C6—C7	1.366 (3)
O1—H1	0.8200	C6—H6	0.9300
O2—C1	1.245 (2)	C7—H7	0.9300
O3—C3	1.354 (2)	C8—C9	1.501 (3)
O3—H3	0.8200	C9—C10	1.377 (3)
O4—C8	1.215 (3)	C9—C14	1.386 (3)
N1—C8	1.365 (3)	C10—C11	1.386 (3)
N1—C5	1.409 (2)	C10—H10	0.9300
N1—H1A	0.8600	C11—C12	1.365 (4)
C1—C2	1.458 (3)	C11—H11	0.9300
C2—C3	1.399 (3)	C12—C13	1.369 (4)
C2—C7	1.404 (3)	C12—H12	0.9300
C3—C4	1.392 (3)	C13—C14	1.377 (3)
C4—C5	1.382 (3)	C13—H13	0.9300
C4—H4	0.9300	C14—H14	0.9300
C5—C6	1.398 (3)		
			
C1—O1—H1	109.5	C6—C7—H7	119.6
C3—O3—H3	109.5	C2—C7—H7	119.6
C8—N1—C5	128.08 (19)	O4—C8—N1	122.7 (2)
C8—N1—H1A	116.0	O4—C8—C9	120.6 (2)
C5—N1—H1A	116.0	N1—C8—C9	116.6 (2)
O2—C1—O1	121.71 (18)	C10—C9—C14	118.9 (2)
O2—C1—C2	122.3 (2)	C10—C9—C8	124.8 (2)
O1—C1—C2	115.96 (18)	C14—C9—C8	116.4 (2)
C3—C2—C7	118.20 (18)	C9—C10—C11	120.3 (2)
C3—C2—C1	120.46 (18)	C9—C10—H10	119.8
C7—C2—C1	121.3 (2)	C11—C10—H10	119.8
O3—C3—C4	116.45 (19)	C12—C11—C10	120.1 (2)
O3—C3—C2	122.51 (17)	C12—C11—H11	119.9
C4—C3—C2	121.04 (18)	C10—C11—H11	119.9
C5—C4—C3	119.5 (2)	C11—C12—C13	120.1 (2)
C5—C4—H4	120.3	C11—C12—H12	120.0
C3—C4—H4	120.3	C13—C12—H12	120.0
C4—C5—C6	119.99 (18)	C12—C13—C14	120.2 (3)
C4—C5—N1	122.9 (2)	C12—C13—H13	119.9
C6—C5—N1	117.12 (19)	C14—C13—H13	119.9
C7—C6—C5	120.4 (2)	C13—C14—C9	120.4 (2)
C7—C6—H6	119.8	C13—C14—H14	119.8
C5—C6—H6	119.8	C9—C14—H14	119.8
C6—C7—C2	120.9 (2)		
			
O2—C1—C2—C3	−1.0 (3)	C3—C2—C7—C6	−0.9 (3)
O1—C1—C2—C3	178.68 (19)	C1—C2—C7—C6	178.3 (2)
O2—C1—C2—C7	179.8 (2)	C5—N1—C8—O4	2.4 (4)
O1—C1—C2—C7	−0.5 (3)	C5—N1—C8—C9	−178.3 (2)
C7—C2—C3—O3	−179.9 (2)	O4—C8—C9—C10	168.2 (2)
C1—C2—C3—O3	0.8 (3)	N1—C8—C9—C10	−11.1 (4)
C7—C2—C3—C4	0.0 (3)	O4—C8—C9—C14	−9.7 (3)
C1—C2—C3—C4	−179.2 (2)	N1—C8—C9—C14	171.0 (2)
O3—C3—C4—C5	−179.1 (2)	C14—C9—C10—C11	−0.8 (4)
C2—C3—C4—C5	1.0 (3)	C8—C9—C10—C11	−178.6 (3)
C3—C4—C5—C6	−1.1 (3)	C9—C10—C11—C12	0.0 (4)
C3—C4—C5—N1	177.6 (2)	C10—C11—C12—C13	0.4 (5)
C8—N1—C5—C4	10.2 (4)	C11—C12—C13—C14	0.1 (5)
C8—N1—C5—C6	−171.0 (2)	C12—C13—C14—C9	−0.9 (4)
C4—C5—C6—C7	0.2 (3)	C10—C9—C14—C13	1.2 (4)
N1—C5—C6—C7	−178.6 (2)	C8—C9—C14—C13	179.3 (2)
C5—C6—C7—C2	0.9 (4)		

### Hydrogen-bond geometry (Å, º)

Cg2 is the centroid of the C9–C14 ring.

**Table:**

*D*—H···*A*	*D*—H	H···*A*	*D*···*A*	*D*—H···*A*
O1—H1···O2^i^	0.82	1.83	2.6470 (19)	176
O3—H3···O2	0.82	1.88	2.601 (2)	146
C4—H4···O4	0.93	2.23	2.828 (3)	122
C12—H12···*Cg*2^ii^	0.93	2.95	3.773 (3)	142

Symmetry codes: (i) −*x*+1, −*y*, −*z*−1; (ii) *x*, −*y*+1/2, *z*+1/2.
