# Supplementary figures and images for: Crystal structure of 4-benzamido-2-hy­droxy­benzoic acid
Source: Acta Crystallogr E Crystallogr Commun. 2015 May 20;71(Pt 6):o409. doi: 10.1107/S2056989015009032 (PMC4459298; doi:10.1107/S2056989015009032)

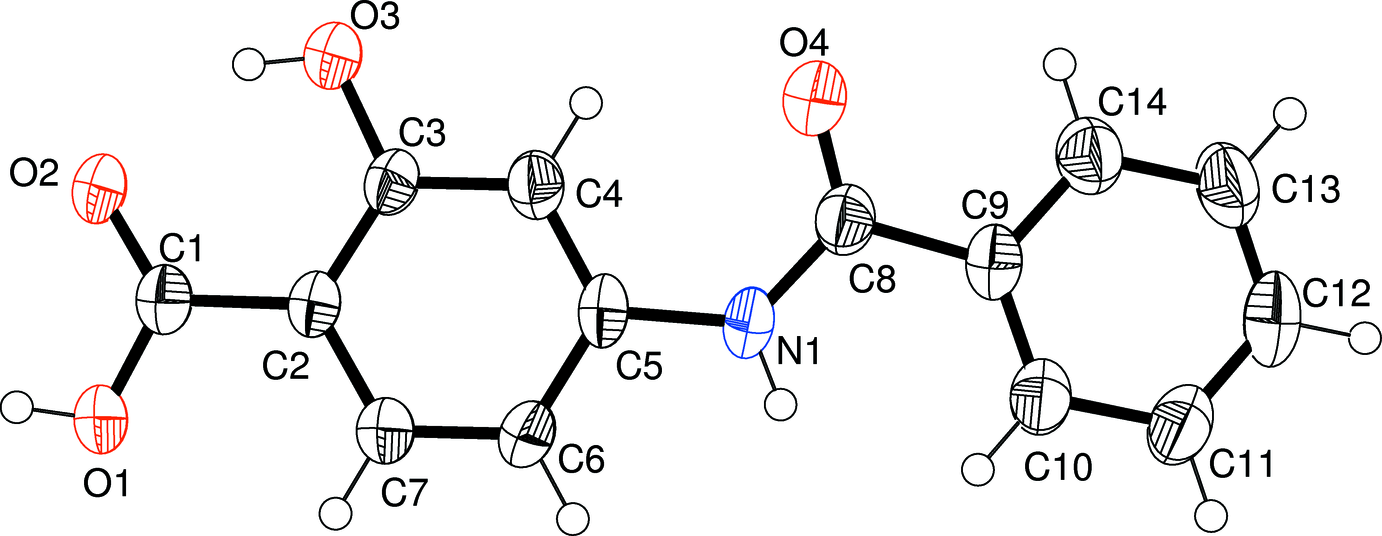

Supplement: Supplementary file 4 [file e-71-0o409-fig1.tif]

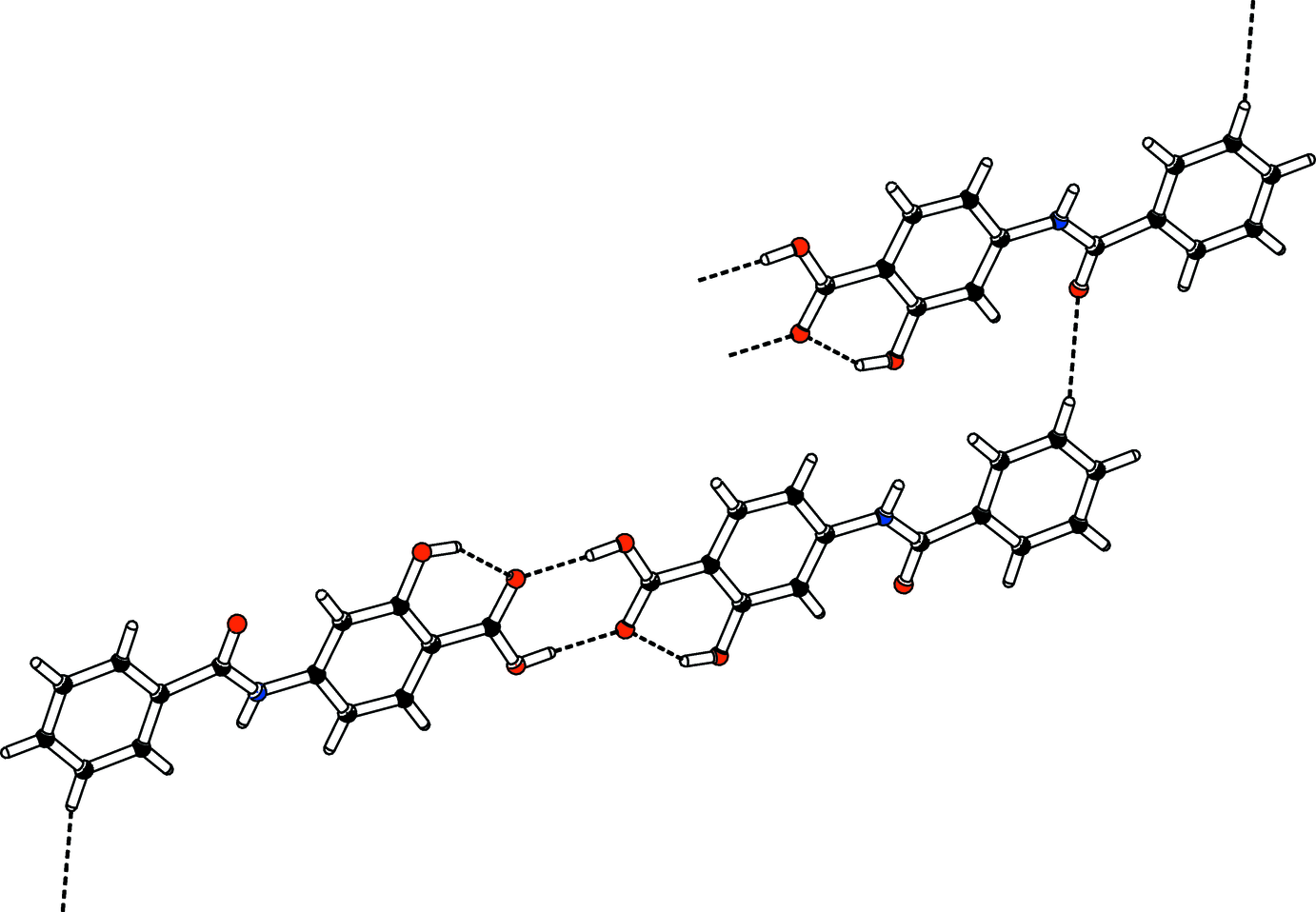

Supplement: Supplementary file 5 [file e-71-0o409-fig2.tif]
